# COVID-19 pandemic: multilevel dental technical guidelines based on new scientific evidence

**DOI:** 10.31744/einstein_journal/2022AE6307

**Published:** 2022-03-07

**Authors:** Sérgio Araújo Andrade, Raissa Emanuelle Lima, Fernando de Pilla Varotti, Omar Abdelwahab, Bashir Abdulgader Lwaleed

**Affiliations:** 1 Universidade Federal de São João del-Rei Divinópolis MG Brazil Universidade Federal de São João del-Rei, Divinópolis, MG, Brazil.; 2 Universidade de Itaúna Itaúna MG Brazil Universidade de Itaúna, Itaúna, MG, Brazil.; 3 University of Southampton Southampton SO United Kingdom University of Southampton, Southampton, SO, United Kingdom.

**Keywords:** Coronavirus infections, COVID-19, SARS-CoV-2, Pandemics, Betacoronavirus, Guideline, Dentistry, Dental care, Containment of biohazards, Telescreening, medical, Personal protective equipment

## Abstract

The COVID-19 pandemic imposed restrictive measures on dentistry in different regions of the world, ranging from stoppage of care to only permission for urgent and emergency dental services. Thus, new biosafety guidelines for resuming activities, whether in single dental offices, large clinics or dental education activities, are urgently required. In this sense, herein, guidelines that incorporate common points of the main protocols found in the literature for the resumption of dental activities at their different levels, whether in the scope of care or education, are presented. Furthermore, we present the incorporation of measures that allow an increase in the level of biosafety, such as the control of the dental team, the inclusion in the history of conjunctivitis as a possible alert for COVID-19, and the use of the pulse oximeter to assess the risk of silent hypoxemia, which may indicate a complication of COVID-19. In addition, new perspectives for directing research and innovation for biosafety in dentistry are discussed.

## INTRODUCTION

Undoubtedly, March 11, 2020 will be remembered for generations to come, when the World Health Organization (WHO) declared coronavirus disease 2019 (COVID-19) as a pandemic.^(1)^ In this context, governments around the world imposed strict measures to contain the spread of the outbreak, such as total lockdown.^(2)^ Thus, there were countries or regions that completely closed dental offices, except for urgent and emergency care.^(3–5)^ Thereupon, due to the need to reopen dental services, guidelines were established by regulatory agencies, class entities or suggested in the scientific literature, considering specific nuances of dental care and its risks in the context generated by severe acute respiratory syndrome coronavirus 2 (SARS-CoV-2).^(5–10)^ However, these protocols for urgent and emergency care or the resumption of activities in dentistry should only be considered as a quick response and not as a broad and definitive solution.^(4,11)^ Thus, such protocols must be constantly improved in order to not only provide solutions for a single viral type, but also to make the profession predictably safe.

Herein, we present the principal converging regulations for resuming dental care, discussing the main impacts of these protocols, outlining perspectives for future research related to these biosafety measures imposed by COVID-19.

## IMPACTS OF COVID-19 ON DENTISTRY: A COMPREHENSIVE PERSPECTIVE

When proposing measures that impact or interrupt dental services, the healthcare regulatory agencies or class entities, need to consider oral health as an integral part of general health, with major impacts on the well-being and quality of life of the population. In this sense, dental caries and periodontal diseases have a progressive character, being among the most prevalent diseases with high potential for morbidity related to pain, infection and loss of function, which can lead to loss of teeth with its adverse psychosocial impacts.^(7,12,13)^ In contrast, oral cancer has high morbidity and mortality rates, affecting 500,550 people with 177,384 deaths, in 2018, and its prognosis is severely affected by the delay in diagnosis.^(12–14)^ Hence, it must be considered that measures that propose the suspension of dentistry activities contribute to a greater probability that oral diseases will evolve, causing events with a greater risk of emergency or urgency, resulting in higher complexity and treatment costs.^(7)^ Furthermore, the diagnosis of oral cancer depends on physical examination, which, is impossible to be performed by teletriage and teledentistry.^(6,9)^ Therefore, the early diagnosis of oral cancer must be highly prioritised to prevent the disease from progressing causing serious and irreversible damage to the patient.^(11,14–16)^

It is noteworthy that dental workers are among the highest risk groups regarding the exposure to SARS-CoV-2, both for acting in close contact with the patient's upper airways, and the possibility of contamination by aerosol and droplets generated during procedures, contaminated specimens or fomites.^(3,5,6,8,9,11,17–19)^ In this context, guidelines from the Centers for Disease Control and Prevention (CDC), American Dental Association (ADA), National Health Service (NHS) and Occupational Safety and Health Administration (OSHA), have become the main references guiding practice of dental care during the pandemic.^(6,7,11)^

Thus, guidelines for resuming appointments or maintaining urgent and emergency dental services have been categorized into: engineering control measures, patient management, face-to-face care protocols and instructions for continuing dental education.^(19)^ However, it should be emphasised that it is up to the local health authorities to define and adjust the scope of the measures to be followed to resume dental services based on the epidemiological and socio-economic complexity of each region.^(11)^

## PERSPECTIVES AND PRELIMINARY GUIDELINES REGARDING SAFEGUARD OF DENTAL STAFF AND ENGINEERING CONTROL

Table 1 summarises the main recommendations before face-to-face care in both the official guidelines and in scientific literature about COVID-19.^(3,4,6–9,11,19)^

**Table 1:** Recommendations prior to face-to-face appointment

Advices	Description
Dental staff controls	Dental workers with comorbidities (hypertension, diabetes, cardiorespiratory and/or cerebrovascular diseases, and cancer) and/or elderly should be removed from the frontline of clinical care, undertaking only administrative activities or teletriage^(4,8)^
	Dental workers with suspected or confirmed case of COVID-19 should be removed from the clinical frontline and kept under medical care^(3,6)^
	Asymptomatic dental workers should be monitored and tested for COVID-19^(3,6,11)^
Engineering controls	Installation of physical barriers that maintain a minimum distance of 2m between people, whether they are patients and/or professionals^(6,8,11)^
	Adequate ventilation with air flow directed from clean to less clean^(3,6,8)^
	Allow 20 to 30 minutes between consultations to ensure sufficient time for cleaning, disinfection, sterilisation and renewal of ambient air. In the waiting room, social distancing should be observed and strictly adhered to, magazines should be removed, as well as ornaments and toys and, if possible, avoid the presence of escorts. Intelligent scheduling for the patient to arrive at the dental office and be cared for “just in time”^(4,6–9,11,19)^
	Preferably, use an individualised dental office. In the case of dental clinics with a large number of chairs, a spacing between chairs of at least 2m should be promoted, which must be positioned parallel to the air flow, with physical barriers between chairs. If available, consider the use of a portable HEPA air filtration unit in all types of dental offices^(6)^
	Use of plastic film in handle-able locations (*e.g*. door handles, equipment hand-pieces, handles of reflectors and keyboards)^(7,19)^
	Avoid the use of a spittoon, prioritising, if possible, the use of high volume suction to minimise aerosol^(3,6,7,11,19)^

HEPA: high efficiency particulate arrestance.

In the context of the proposed measures in table 1, dental workers carry a major and indispensable responsibility to safeguard themselves, individuals and the community. Dental workers are highly susceptible to occupational diseases and, especially the elderly and/or those with comorbidities, should be placed among high-risk groups to develop serious complications or die due to COVID-19.^(4,20–24)^ Thus, public policies are urgently needed to sufficiently address the need of high risk professionals, who may need to take a break from their jobs to avoid possible serious illnesses as a result of COVID -19 pandemic.

Furthermore, COVID-19 pandemic provided the opportunity for individuals to think and innovate to improve biosafety in dentistry, including architectural aspects, patient flow, ventilation and aerosol minimisation, either by controlling its dispersion or using new dental techniques that would not allow the production of the aerosol.

## PERSPECTIVES AND GUIDELINES FOR A FACE-TO-FACE DENTAL CARE FLOW

Table 2 shows the steps and the main characteristics related to the new flow required for resuming dental services during COVID-19.^(3,5–9,11,19,25–32)^

**Table 2:** Steps and the main characteristics related to the new flow required for resuming dental services during COVID-19

Stage	Measures applied
Telephone or video conference triage	Perform anamnesis for assessment of the patient for the presence of signs and symptoms of COVID-19, with careful tracking of all professionals who were in contact with a COVID-19 positive individual.^(3,5–9,11,19)^ In this context, attention should be drawn to the suspicious signs of COVID-19: fever >37.8°C, conjunctivitis, chill, cough, shortness of breath, fatigue, muscle or body pain, headache, loss of taste or smell, sore throat, congestion or runny nose, nausea, or vomiting, diarrhea, which appear 2 to 14 days after exposure to the virus. The presence of indicative signs of severity for COVID-19 should be assessed, including breathing difficulties, persistent pain or pressure in chest, confusion, difficulty keeping awake and bluish lips^(3,6,9,25,31,32)^
	Check the real need for a face-to-face consultation, through a history taking focused on the basic concepts of: – Emergency: situations involving the risk of death of the patient, such as airway obstruction and uncontrolled hemorrhage – Urgency: refers to cases of severe pain and/or infection – Elective procedure: refers to the scheduled consultation where there is no setting that requires emergency^(3,6,7,9,11,26)^
	If available, use teledentistry, which includes remote patient assessment and medication prescription. This remote prescription includes analgesics, anti-inflammatory and/or antibiotics, according to the disease condition, patient preferences, possible complications, and the possibilities coming from the local public health departments^(5,11)^
	Special attention should be given to elderly individuals and/or with comorbidities: if asymptomatic for COVID-19, only urgent or emergency care should be prioritised, and, if available, the possibility of home care should be considered. In turn, if infected with COVID-19, the elderly patient and/or with comorbidities should be referred to a hospital to assess the risk and benefit of urgent or emergency dental care^(11)^
	History-taking should be repeated for each new patient consultation request^(11)^
Care in the face-to-face appointment	Patient must wear a face mask and perform hand hygiene with soap or alcohol 70°^(3,6,7,9,11)^
	Measure temperature, and, if >37.8°C, consider fever^(6,7,9,19)^
	Check oxygen saturation levels with pulse oximeter and, if <93%, refer to medical care due to the risk of silent hypoxemia^(27–29)^
	Repeat the triage history for COVID-19 before appointment^(5,11)^
	Consider the use of a rapid test for COVID-19 since it has high specificity and sensitivity^(6)^
	Use of FFP2, N95 or FFP3 respirators for procedures with no generation of aerosols. However, in suspected or confirmed cases of COVID 19 and/or for any aerosol-generating procedure, it is ideal to use respirators, FFP3, N99 or N100 without exhalation valves^(3,6,8,11,19,30)^
	Priority should be given to emergency and urgent care, postponing elective procedures, avoiding adverse impacts on the patients’ conditions due to delayed care. If possible, carry out the entire treatment in a single consultation^(3,6,9,11,19)^
	Preferably, perform minimally invasive procedures, such as using excavators, chemical agents for tooth decay removal, and rubber dam to minimize the spread of microorganisms^(3,5,7,8,11,19)^
	Avoid procedures that produce aerosol (*e.g.* dental turbines, micro motor hand piece, ultrasonic scalers, air/water syringe and sodium bicarbonate powder jet), or procedures that lead to increased salivation, retching or coughing, such as an intraoral radiographic examination and impression. Otherwise, tomography or extra oral radiographic technique should be used^(3,5–8,11,19)^
	Use PPE, such as goggles, face shields, gloves, apron, long-sleeved disposable fluid repellent coverall, surgical cap and, foot cover, in addition to adoption of work uniform (consider washing the uniform using the hospital services or specialised laundry facilities).^(3,6–9,11,19)^ Change PPE at each appointment to avoid cross-contamination and spread of infection^(7,11)^

PPE: personal protective equipment.

In the context of teletriage, it is necessary to take the history to cover the need for dental intervention against the risk of COVID-19 infection, mainly with regards to the general health status of the patient. Therefore, social distancing must be strictly applied with special preparations for the high-risk patient groups, such as the elderly and those suffering chronic diseases, who might suffer serious life-threatening condition, on top of COVID-19 infection. Since hypoxemia can be silent in some patients, measuring oxygen saturation, which is part of the first protocol, is an important screening tool for conditions that may potentially predispose to severe complications when combined with COVID-19 infection.^(27,28)^ Likewise, we have included conjunctivitis as an important symptom because it may represent the only clinical sign of COVID-19. In addition, due to the dentist working close to the eye region, the need to use goggles and face shield is evident.^(25,31,32)^

In relation to the personal protective equipment (PPE), despite the scarcity of resources, robust scientific evidence regarding the reuse of PPE in a safe way is currently unavailable. Therefore, the current recommendation is to change these after each patient.^(33)^ The recently developed health and safety guidelines in the dental field seem to be directed only towards the prevention of a single viral type, ignoring the provision of protective measures against biological hazards other than COVID-19. Importantly, the presence of viable SARS-CoV-2 in aerosol and on copper has been reported for 3 hours, on wood and fabrics for at least 24 hours, on glass for 48 hours, on stainless steel and plastic for 96 hours, and on external surface of a surgical mask for up to 7 days.^(34,35)^ Thus, in the dental environment we have wood (cabinets), stainless steel, copper (electrical components), non-woven fabric for PPE, fabrics (clothing), plastic and glass, where SARS-CoV-2 has different stability.^(19,34)^ Therefore, the strict disinfection and sterilisation of these materials require different disinfection or sterilisation techniques to ensure the proper maintenance of these valuable resources and preventing their damage.^(19)^ Furthermore, a new design of dental equipment is necessary to minimise the exposure of different materials to contact with biological samples, mainly from aerosol and droplets, unifying the standards of protection, disinfection and sterilisation.

COVID-19 exposed the inadequacy and defects in the regulations upon which dental biosafety was based.^(3,26)^ The indication of the use of respirators in the guidelines is only superficially justified based on the scarcity of PPE. Thus, if the indication of a given respirator is based on the viral size, we must consider that hepatitis B virus (HBV) with 40nm is much smaller than SARS-CoV-2, measuring 60nm to 140nm, and accordingly, will be more needy of face masks to prevent its transmission than SARS-CoV-2. Otherwise, if the need to use a respirator is based on the risk of the aerosol-mediated viral transmission, it should be noted that, based on the recently accepted hypothesis, the prions can be transmitted by aerosols, which are much smaller than a virus.^(36,37)^ Moreover, the literature describes the possibility of transmission of COVID-19 infection by asymptomatic carriers, which, without testing, implies that everyone must be treated as a COVID-19 patient.^(5,18,38,39)^ Perhaps the optimal solution to a shortage of PPE supplies is to expand production by industries and not reuse. Therefore, non-reusable face masks with the highest level of protection are urgently needed to combat other pathogenic threats besides SARS-CoV-2, especially that there are no solid scientific basis for processes or conducts that guarantee a wide decontamination regarding all pathogen types.^(33,36,37,40)^ Similarly, there is a need to develop reliable rapid tests that allow for massive testing, and can be used by the dentists, knowing that the current reverse transcription polymerase chain reaction (RT-PCR) COVID-19 test is not feasible for immediate results and subsequent management actions.^(4,5,41)^

## MULTILEVEL PERSPECTIVES AND GUIDELINES FOR DENTISTRY EDUCATION

Table 3 indicates the main guidelines for continued education in dentistry.^(3,4,11,26,42)^

**Table 3:** The main guidelines for continued education in dentistry

Modality	Features
Theoretical classes	Maintaining remote on-line activities, preferably with live classes to promote students/lecturers interaction^(3,26)^
	Use of education tools, such as participation in on-line seminars, case-based discussions or problem-based learning, and clinical videos^(26)^
Laboratory classes	Pre-clinical didactic videos^(26)^
	Use of PPE suitable for staff and students. Maintaining social distance of 2m, imposing physical barriers and insuring appropriate levels of air flow^(26)^
	Use of virtual reality tools, if available^(26)^
General measures	Insuring that preventive and therapeutic psychological support are in place for students against psychological stress, anxiety and fear caused by the uncertainties arising from the COVID-19 pandemic^(3,26)^
Clinical practices	It should be understood that the techniques of provision of the clinical care is the main component in the training of students in dentistry.^(26)^ Thus, these activities can be postponed, but preparations should be made to deliver sufficient clinical training in an optimal way when it is safe to do so
	It should be acknowledged that there is no way that virtual, on-line or remote tools can replace face-to-face clinical care activities^(26)^
	Consider the recommendations of PPE, engineering controls, proper social distancing and patient flow for cases of clinics with multiple chairs
	Whenever possible, face-to-face urgent and emergency care should be considered. Special measures should be put in place in case of infected or suspected patients with COVID-19, ensuring strict social distancing and anti-infection measures are taken. Another technique worthy of implementing is to divide the patients into groups based on the presence or absence of aerosol generation. They then should be directed into different areas within the practice room. The separation of patients should minimise the risk of spread of COVID-19 as well as enable tracking in case of contamination^(3,26)^
	Similarly, the service teams should be separated to reduce the risk of infection and to facilitate contact tracking in the event of contamination. Thus, a lecturer should be kept in tutoring a specific and reduced number of students^(11)^
	The temperature should be checked and history taken daily, regarding the signs and symptoms of COVID-19, maintaining confirmed and suspicious cases, as well as professors, students and patients from risk groups at home and, when necessary, under medical care^(11)^
	Consider using rapid tests for COVID-19 on staff and patients^(26)^
Continued education	Strong migration trend from traditional conferences to webinars and on-line courses^(4,42)^
	Use of on-line tools that allow audiovisual interaction in real time^(42)^

PPE: personal protective equipment.

Dental education was also massively affected by the COVID-19. In this scenario, the resumption of lecturer/student interaction in a virtual environment is extremely important. Furthermore, resumption of laboratory activities requires basic measures of biosafety/risk assessment, and social distance. However, restart of essential clinical activities for training depends on the behaviour of the pandemic in each region, and must be carefully weighed and planned for in order to guarantee the safety of all involved, be them employees, dental staff and patients. All measures essential to the proper and safe functioning of dental services must be readjusted and strictly applied to the current clinical practices environment. Thus, in the context of continued education, there should be a reduction in courses and face-to-face sessions due to measures of social distancing, which, should be partially replaced by webinars and online courses. Nevertheless, mechanisms for validating the information presented in these webinars must be defined, since many have dubious quality and are based on anecdotal evidence.^(4)^

Notoriously, the COVID-19 pandemic has an ambiguous character for dentistry. An example of a negative impact of the COVID-19 pandemic in dentistry is upon determination of suspension or reduction of services and, the increase in the level and number of required biosafety artefacts, with consequent scarcity and increased costs of PPE. This will negatively impact the economic feasibility of the profession.^(4,17,19,43)^ On the other side, positively, the pandemic exposed the fragility of the currently prevailing biosafety rules, which will gradually require the generation of new knowledge and adjustment. In addition, the need for rapid testing of the population, can open new opportunities for dentistry, due to the ease of collecting salivary samples. Therefore, there are various questions related to the anti-infection measures, which needs to be addressed through robust scientific research. This includes the need for answers regarding the feasibility of PPE reuse, decontaminating or sterilising while maintaining normal functionality with no occupational health risks, and cross-transmission of infection while seeking to save the expensive and scarce PPE. Another question to address is which paths should be followed to reduce aerosol generation, and if there are possible new less invasive techniques or devices that capture and decontaminate the aerosol. Also, what is the ideal configuration of a dental chair and peripheral dental equipment to standardise and optimise the cleaning and decontamination measures? Importantly, what is the ideal architecture and temporal flow of patients that allows for optimal biosafety, and what is the ideal level of a respirator in dental practice? In fact, COVID-19 showed that dentistry was spending most of its resources on knowledge and innovation of products, techniques and procedures, while forgetting to innovate and develop a safe work environment for professional practice.

## CONCLUSION

The drastic effects that COVID-19 imposes on the dentistry profession can be the strongest motivation that may lead to the development of definitive and innovative solutions for a true biosafety in dentistry.
